# Limb loading enhances skill transfer between augmented and physical reality tasks during limb loss rehabilitation

**DOI:** 10.1186/s12984-023-01136-5

**Published:** 2023-01-27

**Authors:** Christopher L. Hunt, Yinghe Sun, Shipeng Wang, Ahmed W. Shehata, Jacqueline S. Hebert, Marlis Gonzalez-Fernandez, Rahul R. Kaliki, Nitish V. Thakor

**Affiliations:** 1 Department of Biomedical Engineering, The Johns Hopkins University, Baltimore, USA; 2 Department of Electrical and Computer Engineering, Tufts University, Medford, USA; 3 Division of Physical Medicine & Rehabilitation, University of Alberta, Edmonton, Canada; 4 Department of Physical Medicine and Rehabilitation, The Johns Hopkins University, Baltimore, USA; 5 Infinite Biomedical Technologies, Baltimore, USA

**Keywords:** Upper-limb prostheses, Myoelectric control, Augmented reality, Proprioception

## Abstract

**Background:**

Virtual and augmented reality (AR) have become popular modalities for training myoelectric prosthesis control with upper-limb amputees. While some systems have shown moderate success, it is unclear how well the complex motor skills learned in an AR simulation transfer to completing the same tasks in physical reality. Limb loading is a possible dimension of motor skill execution that is absent in current AR solutions that may help to increase skill transfer between the virtual and physical domains.

**Methods:**

We implemented an immersive AR environment where individuals could operate a myoelectric virtual prosthesis to accomplish a variety of object relocation manipulations. Intact limb participants were separated into three groups, the load control (CG_LD_; $N=4$), the AR control (CG_AR_; $N=4$), and the experimental group (EG; $N=4$). Both the CG_AR_ and EG completed a 5-session prosthesis training protocol in AR while the CG_LD_ performed simple muscle training. The EG attempted manipulations in AR while undergoing limb loading. The CG_AR_ attempted the same manipulations without loading. All participants performed the same manipulations in physical reality while operating a real prosthesis pre- and post-training. The main outcome measure was the change in the number of manipulations completed during the physical reality assessments (i.e. completion rate). Secondary outcomes included movement kinematics and visuomotor behavior.

**Results:**

The EG experienced a greater increase in completion rate post-training than both the CG_AR_ and CG_LD_. This performance increase was accompanied by a shorter motor learning phase, the EG’s performance saturating in less sessions of AR training than the CG_AR_.

**Conclusion:**

The results demonstrated that limb loading plays an important role in transferring complex motor skills learned in virtual spaces to their physical reality analogs. While participants who did not receive limb loading were able to receive some functional benefit from AR training, participants who received the loading experienced a greater positive change in motor performance with their performance saturating in fewer training sessions.

## Background

The loss of a limb is debilitating, complicates various aspects of an individual’s daily life, and often results in decreased autonomy [1]. According to recent statistics, nearly two million individuals live with a limb amputation in the United States, 41,000 of which have an upper extremity amputation [2, 3]. Upper extremity amputations result in particularly difficult functional deficits, such as inabilities to perform stable object grasping and decreased dexterity [4]. To overcome these deficits, individuals with limb loss (LL) will often use a prosthesis. A popular paradigm of prosthesis control, myoelectric control, leverages the electromyogram (EMG) signals of the muscles in the residual limb to control the degrees of freedom (DoF) of the prosthetic device [5]. While simple prostheses can be effectively controlled using a two-channel direct proportional scheme [6, 7], more advanced devices with expanded DoFs often require more complicated myoelectric pattern recognition-based controllers [8–10].

Regardless of the myoelectric control method implemented, control of an upper-limb prosthesis can be a mentally and physically taxing task [11]. Prosthesis training regimens are necessary to teach the user how to operate their prosthesis effectively [12, 13]. Traditionally, these training regimens are conducted in collaboration with a clinical team consisting of prosthetists, orthotists, and other rehabilitation professionals. Furthermore, training regimens are often scheduled after an individual receives their permanent prosthesis, approximately 6 months post-surgery [14]. The lag between surgery and prosthesis fabrication is generally the result of waiting for the significant anatomical changes that the residual limb undergoes during the healing process to stabilize [15].

However, this traditional timeline is not as effective as it could be. Previous work has shown that the time at which a prosthetic intervention is introduced post-surgery has a significant effect on long-term rehabilitation outcomes. Malone et al. defined a “golden period” of less than 30 days wherein beginning the prosthesis training process with an individual decreases the time to rehabilitation completion as well as increases the successful rehabilitation rate. Furthermore, early training intervention was shown to be critical in reducing the phantom pain incidence rate in the population studied [16]. Unfortunately, the hypersensitivity of the residual limb during this “golden period” makes traditional, physical prosthesis training regimens difficult to implement.

To circumvent limitations with traditional prosthesis training regimens, augmented reality (AR) has been leveraged as a rehabilitation technology. In AR systems, virtual objects are generated and combined with the real, physical environment of the user. AR boasts several advantages over related modalities (e.g. virtual reality), such as more accurate depth perception due to the incorporation of real world distance cues [17] and a reduced risk of adverse effects like virtual reality sickness [18]. Several rehabilitation systems have been developed wherein the user dons a virtual prosthesis and manipulates virtual objects via a myoelectric control strategy [19–21]. These systems have been shown to be valuable in that they: (1) allow for self-paced, at-home training [22], (2) increase user motivation for habitual practice [23], and (3) can offer real-time feedback on grasp characteristics such as optimal force or aperture [24, 25].

Despite AR prosthesis training having several advantages over traditional methods, the efficacy of AR training is still unclear. Although previous work has shown that some of the skills that are acquired performing virtual object manipulations are useful for physical prosthesis operation [21], the learning and transference of more complex motor skills has shown to be difficult to achieve [26]. In studies of individuals with motor deficits, it has been found that the spatiotemporal characteristics of reach-and-grasp movement kinematics vary significantly between virtual and physical environments [27–29]. While some of this discrepancy may be due to a general unfamiliarity with virtual environments, this gap may also be due to differences in sensorimotor cues between virtual simulations and their real-life analogs [30–32]. For example, Rohrbach et al. showed in an object lifting task that while an AR object’s visual cues initially dominate cues from the physical environment and prior cognitive knowledge about grasp parameters such as force, these sensorimotor predictions are discarded when conflicting with cues from other senses such as proprioception [33]. Furthermore, functional imaging studies have shown significant differences in activation profiles in sensorimotor areas of the brain when comparing virtual reality movement tasks to their real-life counterparts [34, 35].

Therefore, an avenue to enhance the effectiveness of virtual environment-based rehabilitation is to increase the sensory fidelity of such systems. When operating in augmented reality, both tactile and proprioceptive senses are uncoupled from the virtual interactions displayed visually. Previous studies have shown that by providing haptic feedback, both user immersion and performance in rehabilitation regimens increase due to the provided sense of touch during virtual object interactions [36–38]. Feedback on the position, motion, or loading of a joint (commonly referred to as “proprioception”) is a less-explored modality in this context [39]. In physical reality, there exists a load on the residual limb while operating a prosthesis due to the mass of the device applied to the socket interface. Chappell et al. showed that by artificially applying this socket force during virtual object interactions, participants become more proficient in dexterous manipulation tasks than if that force is absent [40].

In this work, we investigate the effect limb loading has on skill transfer between augmented and physical reality tasks used in LL rehabilitation. Participants underwent a 7-session longitudinal study wherein they trained the use of their prosthetic limb using traditional muscle training or the Prosthetic Hand Assessment Measure in AR (AR-PHAM) [41–43]. Using the AR-PHAM, participants were asked to complete a variety of dexterous object manipulations in AR with some performing these manipulations with loads applied to the limb controlling the prosthesis. The amount of skill transfer that occurred during their training period was measured via a pre- and post-training assessment using a functional, prosthesis control outcome measure in physical reality. As a complement to the work in Chappell et al., secondary metrics focusing on objective measures of motor strategy execution were computed along with traditional measures of functional task performance.

## Methods

### Augmented reality system overview

**Fig. 1:**
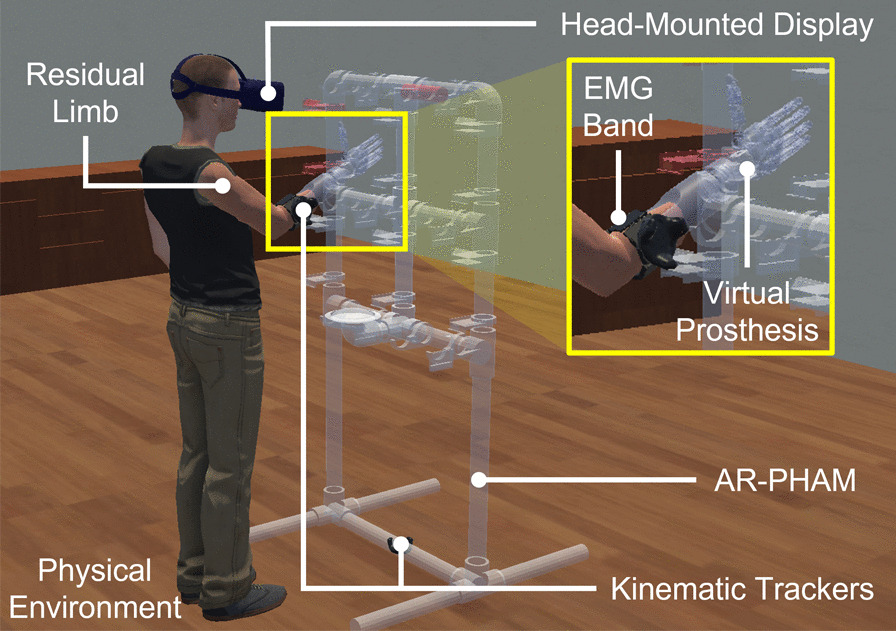
An overview of the augmented reality (AR) system. The head-mounted display (HMD) uses anterior, stereo cameras to pass-through the physical environment to the participant. This pass-through video is combined with virtual representations of a prosthetic limb as well as the AR-PHAM to create the AR scene. Virtual objects are anchored to locations in the physical environment through the kinematic trackers. Participants are tasked to complete a series of object manipulations in this AR environment. To control the virtual prosthesis, participants wear an EMG band on their dominant limb. The resultant EMG signals are then fed into an LDA classifier, translating the EMG data into one of five hand movement classes: rest (RE), hand open (HO), hand close (HC), wrist pronate (WP), or wrist supinate (WS)

The presented AR system allows a participant to operate a virtual upper-limb prosthesis to manipulate virtual objects through the AR-PHAM. The prosthesis extends from a bypass socket worn by the participant on their operating limb. Participants control the virtual prosthesis during rehabilitation tasks using the EMG signals collected from their operating limb. The system also logs task kinematic and electrophysiological data for offline analysis, including limb position, gaze behavior, and raw EMG (Fig. 1).

To render the AR environment, the system uses the VIVE Pro Eye head-mounted display (HMD) (HTC Corporation, New Taipei City, Taiwan). The HMD includes a pair of $640 \times 480$ stereo cameras on the anterior of the headset. These cameras project video of the physical environment into the background of the virtual scene to create an augmented reality view. The HMD also contains internal eye-tracking hardware that allows the system to measure visuomotor behavior such as gaze direction and visual attention (Tobii Technology AB, Stockholm, Sweden).

In the AR environment, participants operate a virtual prosthetic limb modelled after the Modular Prosthetic Limb (MPL) from the Johns Hopkins Applied Physics Laboratory [44]. For the virtual prosthetic limb, we implemented 5 hand movement classes that mirror those available with the physical prosthetic system: rest (RE), hand open (HO), hand close (HC), wrist pronation (WP), and wrist supination (WS).

Virtual objects, such as the virtual prosthetic limb and AR-PHAM are anchored to locations in the physical environment using HTC VIVE trackers. These trackers combine infrared, optical LEDs with inertial measurement units to provide their 3D positions and orientations to the AR system. The AR system uses two of these kinematic trackers: one to anchor the virtual prosthesis to the operating limb and another to anchor the AR-PHAM to the participant’s physical environment. To anchor the virtual prosthesis, a tracker was affixed to a bypass socket attached to the user’s operating limb. To anchor the AR-PHAM, the other tracker was placed at a location on the floor with enough clearance in the physical world.

Surface EMG data acquisition was accomplished using a wireless, eight-channel EMG band (Thalmic Labs, Ontario, Canada). Each electrode recorded bipolar surface EMG signals with a sampling rate of 200 Hz. Features were extracted from the EMG signal with a 250 ms sliding window with 50 ms of overlap. For each channel of EMG, the following time-domain features were extracted: mean absolute value, variance, waveform length, zero crossings, and slope sign change [8]. These features were then used to train a linear discriminant analysis (LDA) classifier for forward prosthesis control [45]. LDA was chosen as the control algorithm for its simplicity and wide availability in commercial pattern recognition systems. Predictions from the LDA classifier were post-processed using a 5-decision uniform vote filter to improve classifier stability.

Kinematic data, such as positions and orientations from the HTC VIVE tracker, was filtered using a 6-Hz second-order Butterworth low-pass filter in both the forward and backward directions, resulting in fourth-order filtering with zero phase-distortion [46]. Gaze data, such as the virtual gaze vector, was filtered using a 10-Hz second-order Butterworth low-pass filter [47].

### Experimental protocol

#### Participants

This study was conducted in accordance with a protocol approved by the Johns Hopkins University School of Medicine Institutional Review Board. Before being tested, each participant gave his or her written informed consent. The participants consisted of twelve individuals with intact limbs (IL). IL participants include 7 males and 5 females and range in age from 18 to 31 years old. All IL participants were right-hand dominant and naïve to pattern recognition myoelectric control. Participants were randomly assigned to either the load control group (CG_LD_; $N=4$) which underwent traditional muscle training, the AR control group (CG_AR_; $N=4$) which underwent no-load AR training, or the experimental group (EG; $N=4$) whose members underwent AR training while simultaneously loading their limb with weight.

#### Physical reality pre- and post-test

Participant prosthesis control proficiency was assessed before and after training using the Prosthetic Hand Assessment Measure (PHAM) [41].

**Fig. 2:**
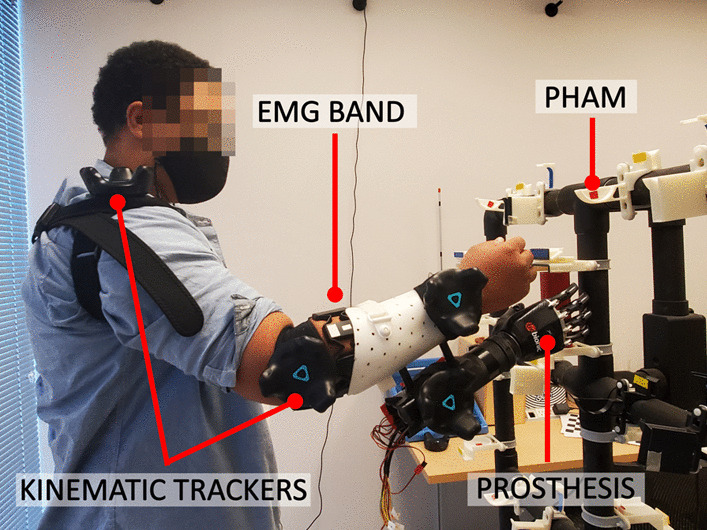
The experimental setup for the physical assessment. Participants use their EMG signals to control a multi-articulated prosthetic hand and wrist attached via a bypass socket. Participants also wear multiple trackers that measure their limb kinematics

For the physical assessment, the user operated a prosthetic system including a bebionic3 prosthetic hand (Ottobock, Duderstadt, Germany) and Motion Control Wrist Rotator (Fillauer LLC, Chattanooga, TN). As mentioned previously, the prosthetic system implements 5 hand movement classes: RE, HO, HC, WP, and WS. An EMG band was placed circumferentially around the forearm of the participant’s dominant limb. The EMG band’s location and orientation were recorded to ensure a similar configuration between the pre- and post-test. Kinematic trackers were also mounted at joint locations on the operating limb: at the shoulder, the elbow, and the wrist. This prosthetic system was incorporated into the bypass socket to allow for end-effector consistency between all participants (Fig. 2).

The physical testing protocol began with training the myoelectric pattern recognition classifier. While wearing the bypass socket, participants were presented with a series of visual cues, each prompting the activation of a specific hand movement class. Movement cues were presented for 5 s, of which EMG data was only collected for the last 3 s. For HO/HC, participants performed the anatomically correct hand movement, while for WP/WS, participants substituted wrist rotation with wrist flexion/extension for easier discriminability by the classifier. Furthermore, participants were instructed to activate each hand movement class with muscle contractions between 60 and 80% of their max voluntary amplitude. This was done to reduce the effect of muscle fatigue on classifier performance during prolonged assessments [48]. Participants were also instructed to perform this process in three limb positions: (1) limb at side with elbow at 90°, (2) limb reaching forward at chest-level with elbow straight, and (3) limb reaching forward above the head with elbow straight. This was done to reduce the effect that different limb positions have on myoelectric control performance [49].

EMG features were then extracted from the collected data and used to train the LDA classifier. Classifier validation was accomplished using a 67/33 train/test split. If the validation accuracy for each movement class was greater than or equal to 80%, the classifier was considered valid, and the participant proceeded to the next stage of validation. If not, the participant repeated the training data acquisition described previously until this threshold was achieved. Preliminary testing and previous results found 80% to be a reasonable threshold since training data was collected in multiple limb positions while the participant wore a bypass socket [50].

In the next stage of validation, participants were allowed to practice operating the prosthetic system for a maximum of 5 min. If the participant was unable to elicit each hand movement class with reliability in a neutral limb posture (limb at side with elbow at 90°), they were given the opportunity to retrain the LDA classifier and restart the validation process. Once the participant felt comfortable controlling the prosthesis, they proceeded to attempt the PHAM.

**Fig. 3:**
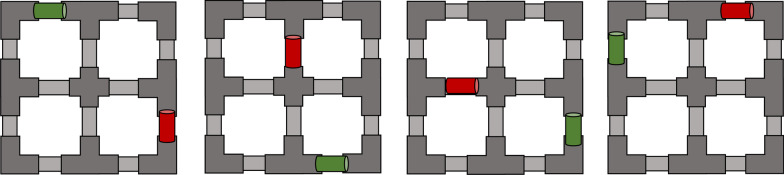
An illustration of the 4 rehabilitation tasks for the physical and augmented reality PHAM. Cylinders will start in an initial location and orientation (red) with the corresponding target location and orientation being highlighted on the PHAM frame (green). Each rehabilitation task requires: a positional change in elevation, a positional crossing of the participant’s midline, and a 90° change in object orientation

The PHAM consists of a series of object manipulations within a participant’s 3D reaching volume. Participants are tasked with transporting a cylinder from one start location on the PHAM’s frame to a prompted end location within a time limit. Each object transport requires 2 DoF, HO/HC and WP/WS. Tasks were considered unsuccessful if the participant dropped the cylinder during manipulation or if the participant was unable to complete the manipulation within the allotted time of 15 s. Participants were asked to complete a total of 4 tasks, 10 times each, prompted in a random order (Fig. 3). Participants were given the opportunity to rest between each task to counteract the effects of muscle fatigue.

#### Augmented reality training

Participants in the CG_AR_ and EG underwent prosthesis training in AR using the AR-PHAM platform. Similar to the physical assessment, participants wore the EMG band around the forearm of their dominant limb. Participants then performed the same myoelectric pattern recognition training and validation protocol as outlined for the pre- and post-tests. Once the classifier was trained, participants were instrumented with the AR-specific hardware, such as the HMD. Participants in the EG also wore the physical prosthesis system on the limb with the EMG band, although it was not operational. This was done to accustom the user to the load of the prosthetic system (1.17 kg).

At the beginning of each training session, participants were allowed to explore and become familiar with the AR environment for a maximum of 10 min. During each training session, participants were asked to accomplish a series of object relocation tasks on the AR-PHAM frame. Participants operated the virtual prosthetic limb via pattern recognition to manipulate cylinders from some initial configuration to some final configuration. Each pair of initial and final object configurations was characterized by 3 properties: (1) a positional change in elevation, (2) a positional crossing of the participant’s midline, and (3) a 90° change in object orientation. With this, each object manipulation task required 2 DoF (HO/HC and WS/WP) and proficient control in a large portion of the user’s reaching space. The tasks performed with the AR-PHAM are the same as those prompted during the physical assessment (Fig. 3).

**Fig. 4:**
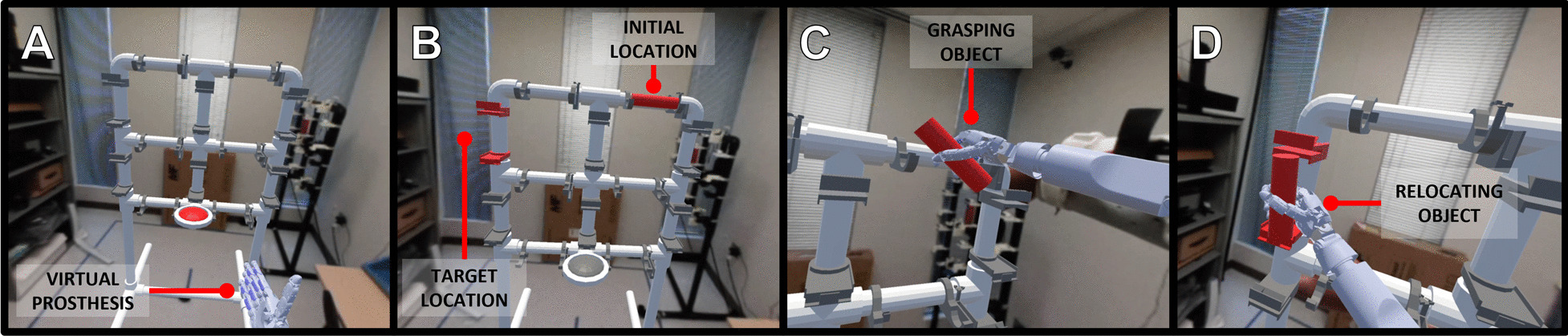
Different stages of a user completing an AR-PHAM rehabilitation task. **a** The AR-PHAM is anchored in the user’s physical environment and prompts the user to begin a task by pressing the central red button. **b** Once started, the task spawns a red cylinder on the AR-PHAM frame and prompts a target location and orientation by highlighting an AR-PHAM object holder red. **c** The user then reaches for and manipulates the cylinder using the virtual prosthesis, controlling the device via their EMG signals and the LDA classifier. **d** The user transports the cylinder to the target location and releases it onto the object holder with the appropriate orientation

Each AR-PHAM task is self-initiated by the user by pressing the AR-PHAM’s central button. Once a task begins, a cylinder appears in 1 of the 4 initial configurations with the corresponding final configuration highlighted on the AR-PHAM frame. To complete a task, participants must reach out with the virtual prosthesis, grasp the cylinder, and transport the cylinder to the prompted destination. Once there, the operator must release the cylinder onto the AR-PHAM frame with the appropriate orientation to trigger the end of the trial (Fig. 4).

Virtual object grasping was mediated by contact points defined on the distal phalanges of the virtual prosthetic limb. When all 5 phalanges made contact with the cylinder, virtual connections were created in the simulation to adhere the cylinder to the fingers. These connections were configured with a break force of 0.5 N, the normal force required to overcome the frictional force of the cylinder [51]. If the participant opened the virtual prosthesis during grasping, the connections broke and the cylinder would begin to slip and fall, until all 5 phalanges made contact with the cylinder again. No explicit visual feedback was provided to the participant on the state of the grasping interaction, so as to maintain parity with the physical assessment. Participants may fail a manipulation task if they exceed the time limit of 15 s or if they drop the cylinder on the floor. Participants were asked to complete each of the 4 motor tasks 10 times for a total of 40 trials.

#### Muscle load training

Instead of AR training, participants in the CG_LD_ underwent a muscle strengthening protocol during the 5-session training period. During each session, participants wore the non-operational physical prosthesis system on their dominant limb. Participants were then asked to perform a series of arm lift exercises. Each arm lift required the participant to raise the prosthesis from a position at their side to a position with their limb reaching forward at chest-level with their elbow straight. Participants were instructed to hold this posture for 15 s before returning the prosthesis to their side, after which they would have 15 s to rest before the next arm lift. Participants repeated this sequence for a total of 40 arm lifts each training session.

### Data segmentation

**Fig. 5:**
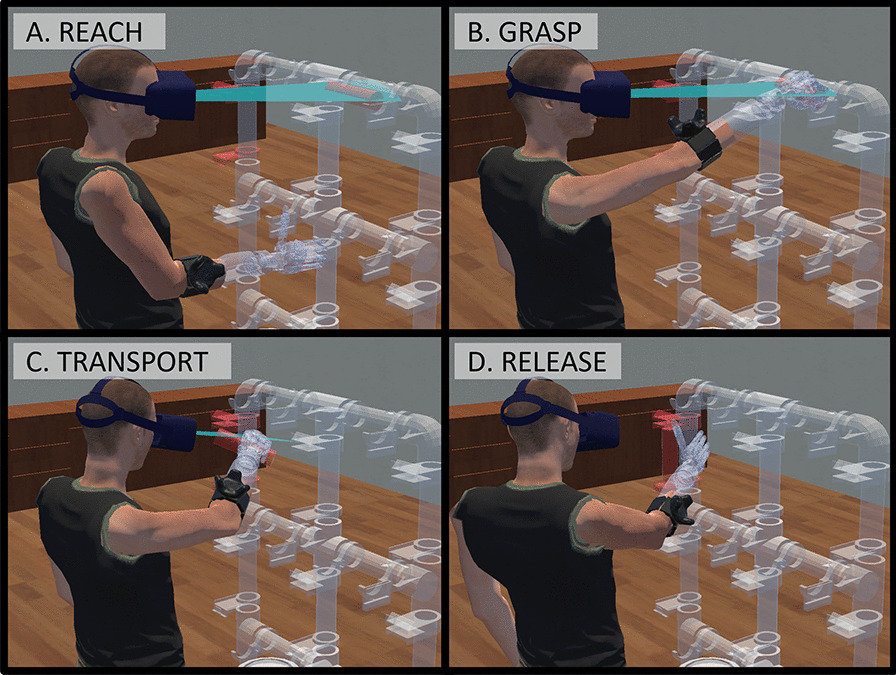
The four phases of a successfully completed AR-PHAM rehabilitation task. **a** Beginning with the *Reach* phase, the user visually focuses the red cylinder and moves the virtual prosthesis into the cylinder’s proximity. **b** Followed by the *Grasp* phase, the user operates the virtual prosthesis via myoelectric control to grab the cylinder. **c** During the *Transport* phase, the user visually focuses the cylinder’s destination and moves the virtual prosthesis to relocate the cylinder. **d** In the final *Release* phase, the user again operates the virtual prosthesis to place the correctly aligned cylinder onto the destination location

Each object manipulation task of the AR-PHAM was segmented into four phases to allow for more granular analysis of a participant’s task motor strategy. These phases are: *Reach*, *Grasp*, *Transport*, and *Release* (Fig. 5). A task began in the Reach phase after the AR-PHAM’s central button was pressed and sequentially progressed through the remaining phases with the following transition criteria:

1. *Reach–Grasp* The prosthesis was within 15 cm of the object’s starting position and the myoelectric control signal output five consecutive, non-rest classifications
2. *Grasp–Transport* The participant had sent at least one HC command to the prosthesis and the prosthesis and the target object were greater than 2 cm from the object’s starting position
3. *Transport–Release* The object was within 5 cm of its target position and moving less than 0.15 m/s

The end of the *Release* phase (and the end of the manipulation task) is defined as when the object is within 2 cm of its target position and 15° of its target orientation and the participant sends a HO command to the prosthesis. All transition thresholds were determined experimentally using preliminary data.

### Data analysis

The main outcome measure reported is the change in the task completion rate for the physical PHAM. Experimental data from the AR-PHAM training period was also analyzed along two secondary dimensions: compensatory motion and visuomotor behavior. Evaluation along these secondary dimensions was used to assess the quality of the movements achieved during virtual object manipulation. Secondary analyses included data only from successfully completed tasks.

Compensatory motion was quantified by computing the angular range of motion (RoM) for the glenohumeral and elbow joints. Of the upper-limb DoF, we computed the RoM for shoulder flexion and extension ($\theta_{\text{FLEX}}$), shoulder abduction and adduction ($\theta_{\text{ABAD}}$), and elbow flexion and extension ($\phi _{\text{FLEX}}$). $\theta _{\text{FLEX}}$ and $\theta _{\text{ABAD}}$ were computed as the angles that the participant’s upper arm ($v_{ua}$, defined as the normalized vector from their shoulder position to their elbow position) made with the anatomical planes of their trunk. The angle the upper arm made with the coronal plane of the participant’s trunk was defined as shoulder flexion (+) and extension (−). The angle the upper arm made with the sagittal plane of the participant’s trunk was defined as shoulder abduction (+) and adduction (−). Equations 1 and 2 were used to compute the magnitude of the glenohumeral angles:

1 $$\theta _{\text{FLEX}}= \arccos \left( v_{ua} \cdot \frac{v_{ua} \cdot \left[ 1 \ 0 \ 1 \right] ^{\top }}{\Vert v_{ua} \cdot \left[ 1 \ 0 \ 1 \right] ^{\top } \Vert } \right)$$

2 $$\theta _{\text{ABAD}}= \arccos \left( v_{ua} \cdot \frac{v_{ua} \cdot \left[ 0 \ 1 \ 1 \right] ^{\top }}{\Vert v_{ua} \cdot \left[ 0 \ 1 \ 1 \right] ^{\top } \Vert } \right)$$

The sign of these angles was determined to be the sign of the component of $v_{ua}$ normal to the projection plane (i.e. *y*-component for $\theta_{\text{FLEX}}$, *x*-component for $\theta_{\text{ABAD}}$).

$\phi_{\text{FLEX}}$ was defined as the angle made between the participant’s upper arm ($v_{ua}$) and forearm ($v_{fa}$, defined as the normalized vector from their elbow position to their wrist position). $\phi_{\text{FLEX}}$ was computed using Eq. 3:

3 $$\phi _{\text{FLEX}} = \arccos \left( v_{ua} \cdot v_{fa} \right)$$

Joint angular RoM was then computed as the difference between the maximum and minimum values along these DoF.

Visuomotor behavior was quantified using the metrics defined by the Gaze and Movement Assessment (GaMA) [47, 52, 53]. The GaMA is concerned with describing the spatiotemporal relationship between prosthesis movement and the user’s visual attention and fixation. For the PHAM object manipulation tasks, the following areas of interest (AOI) were defined: the prosthetic hand, the manipulable object, the object’s starting location, the object’s ending location, and the PHAM’s central button. While GaMA provides a variety of visuomotor metrics to consider, we report the percent fixation time of the object during transport.

Statistical analysis was performed using Python 3.7 and MATLAB R2019b. The non-parametric Mann-Whitney test was used due to the limited sample size. For all metrics, the threshold for statistical significance was set at $p<0.05.$

## Results

### Training EMG validation

To validate the integrity of the initial EMG data used to train the LDA algorithm on each day, an upper bound on the multi-class probability of error ($P_e$) was calculated. $P_e$ refers to the probability that any EMG sample is misclassified by the LDA algorithm and is defined as the minimization of the expected pairwise Bhattacharyya distances (*B*) between the EMG feature distributions defining each possible movement classification (Eq. 4) [54]:

4 $$P_e \le \left( \sum _{i=1}^{N} \sum _{j=1}^{N} \frac{1}{2} \text{e}^{-B_{ij}} \right) - \frac{1}{2}$$

**Fig. 6:**
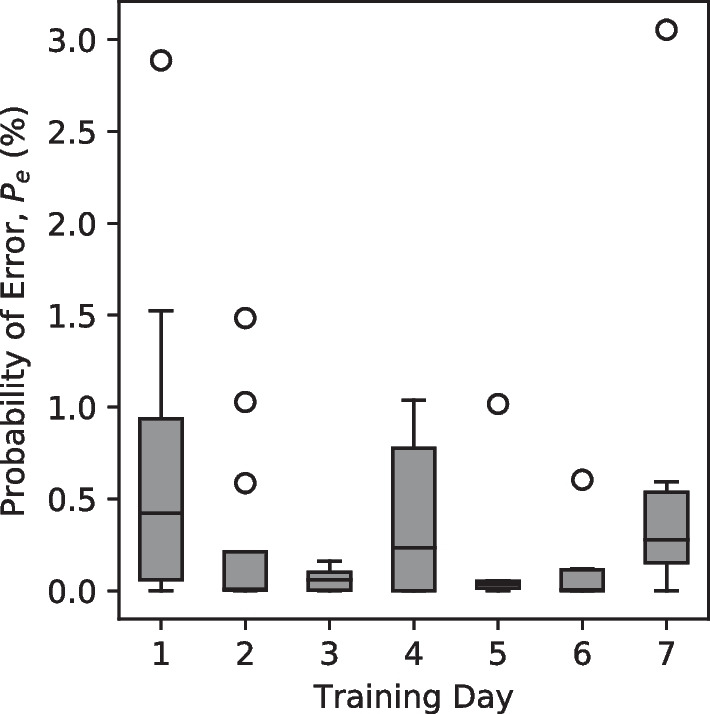
Maximum probability of a classification error ($P_e$) in the initial training set for each session for all IL participants using the LDA algorithm. For all IL participants, $P_e$ did not exceed 4%. Sample size, $N=8$ for days 2 through 6, $N=12$ otherwise

For all IL participants, the multi-class probability of error did not exceed 4% and for most participants, this error did not exceed 1.5% (Fig. 6).

### Physical assessment performance

**Fig. 7:**
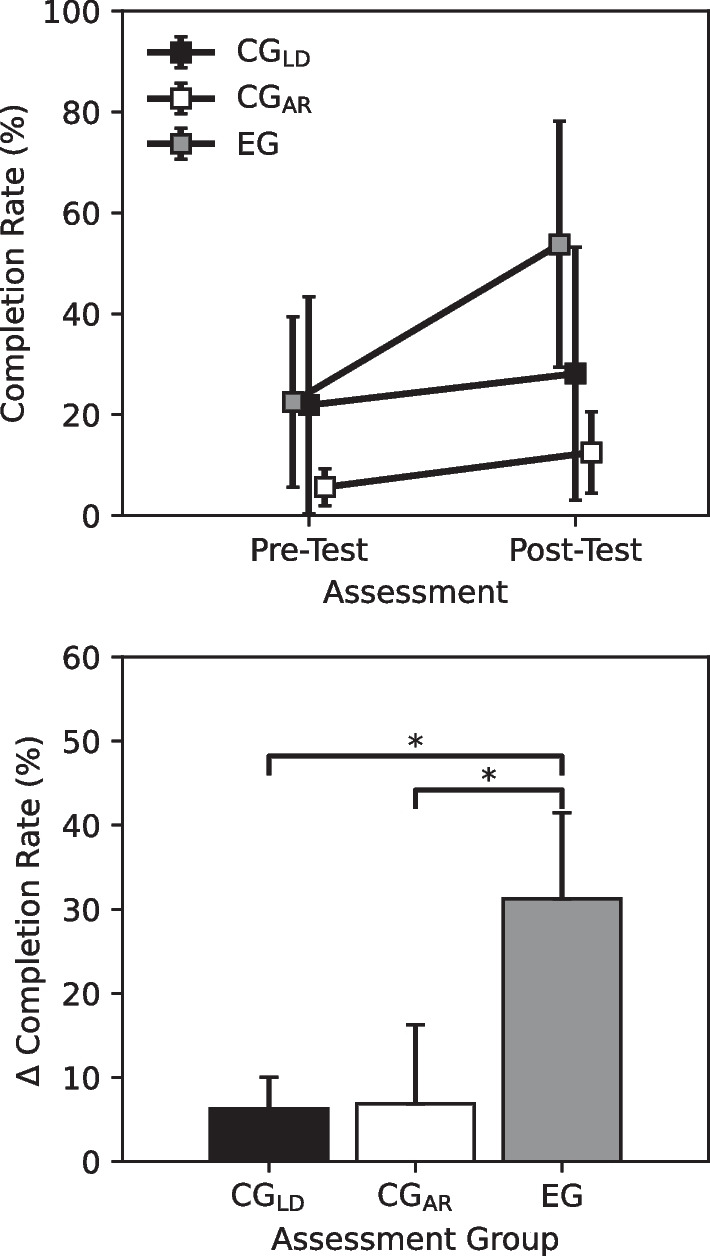
Change in performance between the pre- and post-test PHAM. Participants in the experimental group (EG) experienced an average increase in task completion rate of 31.25%, while participants in the load (CG_LD_) and AR (CG_AR_) control groups experienced an average increase in performance of 6.25% and 6.88%, respectively. Error bars denote standard deviation. *Denotes $p< 0.05$ for the Mann–Whitney Test. Sample size, $N=4$

Participants who underwent the AR training with limb loading experienced a significantly greater increase in completion rate between the pre- and post-test assessment when compared to both participants who underwent the AR training with no limb loading and participants who did simple muscle training ($p<0.05$, Mann–Whitney) (Fig. 7). Participants in the EG experienced an increase in task completion rate of $31.25 \pm 10.23\%$ while participants in the CG_AR_ experienced an increase of only $6.88 \pm 9.42\%$ and participants in the CG_LD_ experienced an increase of only $6.25 \pm 3.75\%$. While all participants in the EG exhibited more functional prosthesis control during the post-test assessment than they did during the pretest assessment, this was not the case for all participants in the CG_AR_. Some CG_AR_ participants reported no change, or even a slight decrease, in performance.

### Augmented reality training performance

**Fig. 8:**
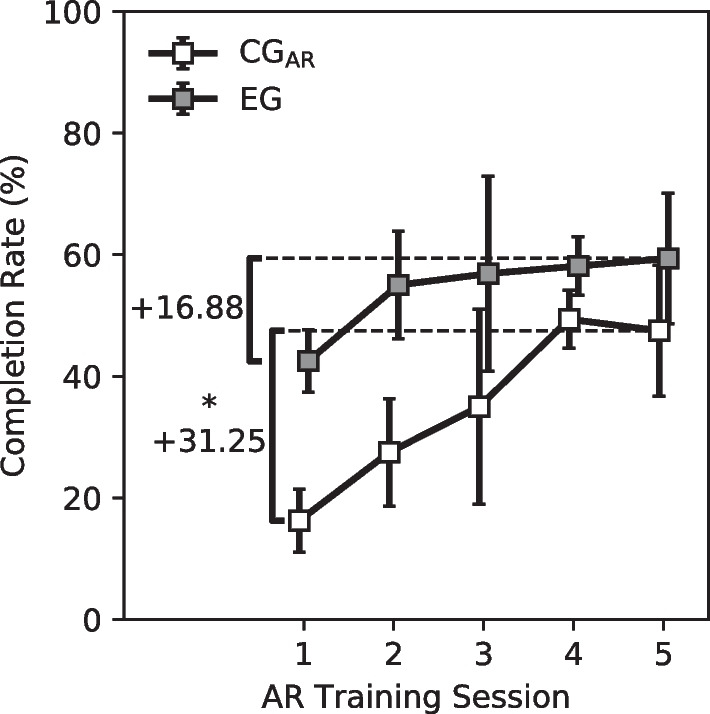
Performance of participants during the AR-PHAM training. Participants in the CG_AR_ experienced an average increase in performance of + 31.25% during training while participants in the EG experienced a + 16.88% increase over the same time period. Despite a greater change in performance, the EG still outperformed the CG_AR_ in the final training session: 59.38% vs 47.5%. Error bars denote standard deviation. *Denotes $p< 0.05$ for the Mann–Whitney Test. Sample size, $N=4$

While training with the AR-PHAM, participants in the CG_AR_ experienced a significant increase of + 31.25% in the average task completion rate between sessions 1 and 5 ($p<0.05$, Mann–Whitney). For participants in the EG, this increase in performance was more mild at + 16.88% (Fig. 8). Both assessment groups seemed to experience a steady increase in functional performance over the first few sessions with performance eventually reaching a steady-state value by the final session. Despite the difference in performance increase, the EG still outperformed the CG_AR_ during the final training session: $59.38 \pm 13.62\%$ (EG) vs $47.5 \pm 10.75\%$ (CG_AR_).

### Joint range of motion

For both the pre-test and the post-test, participants in all groups presented with similar joint RoM. During the pre-test, the CG_LD_ presented with a shoulder flexion range of $69.57 \pm 6.23$°, a shoulder abduction range of $67.89\pm 9.67$°, and an elbow flexion range of $102.62 \pm 25.98$°. This is in comparison with participants in the CG_AR_, who completed pre-test manipulations with a shoulder flexion range of $75.75 \pm 28.38$°, a shoulder abduction range of $60.24 \pm 22.73$°, and an elbow flexion range of $84.72 \pm 39.15$°, and participants in the EG, who completed pre-test manipulations with a shoulder flexion range of $77.77 \pm 7.01$°, a shoulder abduction range of $43.07 \pm 12.12$°, and an elbow flexion range of $71.36 \pm 13.54$°.

During the post-test, the CG_LD_ completed manipulations with a shoulder flexion range of $88.77 \pm 26.89$°, a shoulder abduction range of $72.51 \pm 4.81$°, and an elbow flexion range of $116.00 \pm 10.77$°. Participants in the CG_AR_ completed the same set of manipulations with a shoulder flexion range of $80.04 \pm 30.43$°, a shoulder abduction range of $54.83 \pm 20.40$°, and an elbow flexion range of $77.05 \pm 31.93$°. Participants in the EG completed these manipulations with a shoulder flexion range of $83.87 \pm 20.66$°, a shoulder abduction range of $52.76 \pm 20.56$°, and an elbow flexion range of $88.29 \pm 37.58$°.

**Fig. 9:**
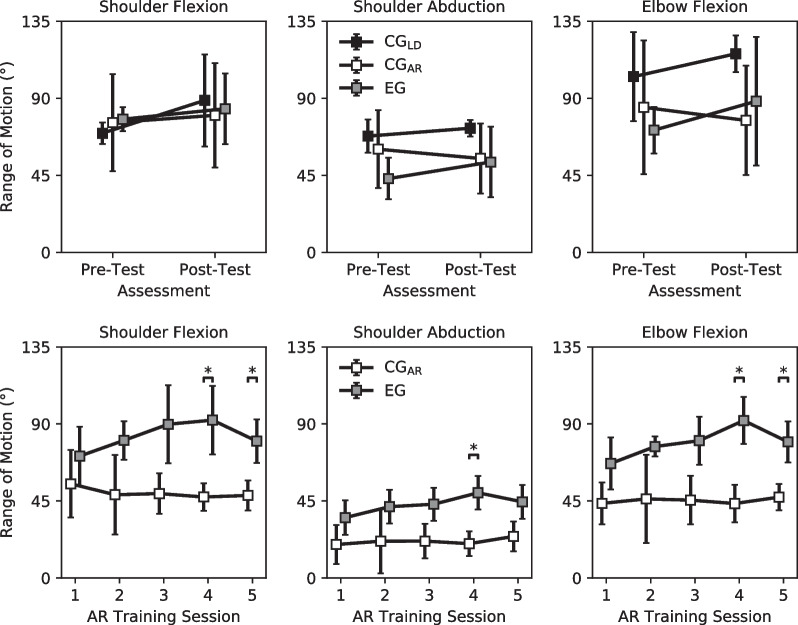
Upper-limb joint range of motion (RoM) during successfully completed tasks. (Top) In both the pre-test and post-test, all IL participants presented similar joint RoM on average for all upper-limb DoF. (Bottom) Across AR training all sessions, the EG presented a greater mean RoM than the CG_AR_ in the following upper-limb DoF: shoulder flexion ($82.34 \pm 33.47$° vs $49.79 \pm 21.36$°), shoulder abduction ($44.38 \pm 20.14$° vs $22.76 \pm 15.02$°), and elbow flexion ($80.80 \pm 29.90$° vs $46.62 \pm 22.21$°). Error bars denote standard deviation. *Denotes $p < 0.05$ for the Mann–Whitney Test. Sample size, $N=4$

During AR training, for each upper-limb DoF of interest, the EG produced a greater average joint RoM when successfully completing an object manipulation when compared to the CG_AR_. For the EG, the average joint RoM produced during successful manipulations over all AR training sessions was $82.34 \pm 33.47$°, $44.38 \pm 20.14$°, and $80.80 \pm 29.90$° for shoulder flexion, shoulder abduction, and elbow flexion. For the CG_AR_, the produced joint ranges were $49.79 \pm 21.36$°, $22.76 \pm 15.02$°, and $46.62 \pm 22.21$°, respectively. Differences in joint RoM were significant in the final two AR training sessions ($p < 0.05$, Mann–Whitney) (Fig. 9).

### Visual fixation percentage

**Fig. 10:**
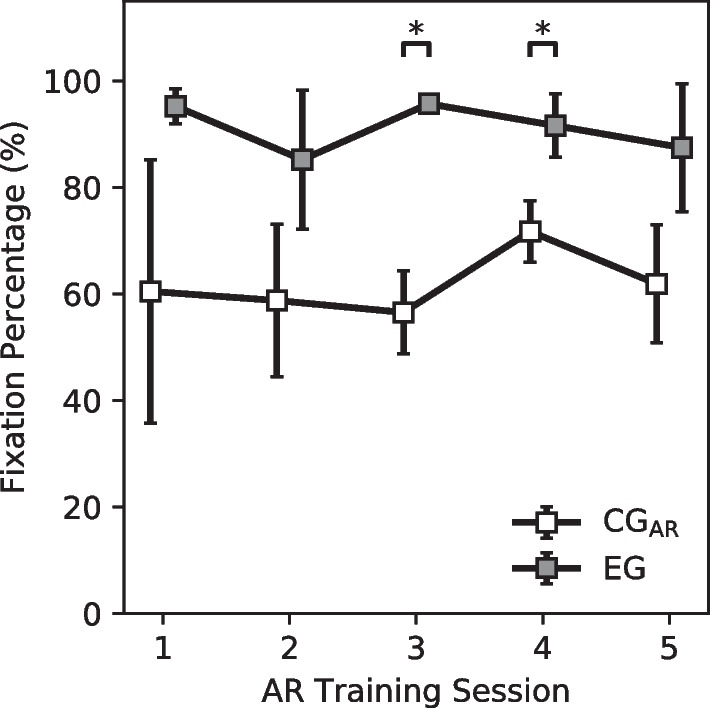
Percentage of time spent visually fixating to the prosthetic hand during object transport during successfully completed tasks. Across all sessions, the EG presented a greater mean fixation percentage ($90.37 \pm 13.32\%$) than the CG_AR_ ($64.09 \pm 23.85\%$). Error bars denote standard deviation. *Denotes $p < 0.05$ for the Mann–Whitney Test. Sample size, $N=4$

The amount of time that an individual fixated on their prosthetic hand during successful task completion in AR was different between the two assessment groups. For the CG_AR_, the average percentage of time spent fixating on the prosthesis was 64.09 ± 23.85% of the total time spent attempting to transport a successfully grasped object. For the EG, the respective average percentage was 90.37 ± 13.32%. While these averages do not seem to meaningfully change throughout the course of the AR training, this trend in visuomotor behavior exists within all 5 training sessions with significant differences existing in training sessions 3 and 4 ($p<0.05$, Mann–Whitney) (Fig. 10).

## Discussion

In this manuscript, we investigate the effect that limb loading has on skill transfer between AR upper-limb prosthesis training and its real-world analog. The results of the three-group longitudinal study show that individuals that undergo a virtual prosthesis training regimen with a load applied to their operating limb experience a greater increase in dexterous functionality with a physical prosthesis post-training than individuals who undergo the same training with no load and individuals who only undergo traditional muscle training (Fig. 7). Additionally, this greater increase in performance is accompanied by a quicker time to saturation for performance in the AR training environment. For participants in the EG, there was no significant difference in their performance between session 1 and session 5 of the AR training period. This suggests that most of the benefit gained during the loaded AR training was immediate, gained after only a single session. This is contrary to the CG_AR_, which required nearly the entire training period to reach steady-state performance (Fig. 8). This difference in saturation time is particularly important for rehabilitation protocols, for which there is a general difficulty in ensuring that patients return for multiple sessions.

The task performance results bring attention to a limitation with not only AR but also physical prosthesis training platforms. Gravity compensation of the prosthesis load during training or evaluation [55–57] is a common concept often used to reduce muscle fatigue during prosthesis operation. However, the results of this study show that the motor skills learned while operating a prosthesis with no loading information do not transfer efficiently to traditional prosthesis operation. This suggests that in order to provide users with the most benefit from their prosthesis training (in terms of transferable performance), protocol designers should avoid the use of gravity compensation techniques.

One important distinction that was reinforced in this study is the unreliability of offline training accuracy as an indicator of online functionality. For all participants, the maximum probability of a classification error in a session’s initial training set was less than 4% (Fig. 6). This is in stark contrast to, for example, the range of task completion rates achieved during the AR-PHAM training: 12.5–67.5% (Fig. 8). Furthermore, this disparity exists despite collecting training data in multiple limb positions both with and without loading the limb. While previous literature suggests this sort of dynamic training can improve the utility of real-world prosthesis use [50, 58], offline validation results remained a poor indicator of prosthesis control functionality for this task. A possible explanation for this could be the “all-or-nothing” nature of the manipulations prompted in this experiment. For example, a participant could be generating the correct movement classifications for the majority of an object manipulation, however, a brief instant of misclassifications may cause the task to become irrecoverable (e.g. dropping the cylinder). It is possible a less strict control task may show better congruence between offline and online performance, but such lax tasks are not indicative of real-world use and so have limited usefulness.

This study also highlights a kinematic difference between accomplishing a motor task with and without the proprioceptive input of a prosthesis load. While previous work has shown that upper-limb prosthesis users produce compensatory movements in the proximal limb joints during object manipulation, these studies suggest that compensatory movements arise primarily from a lack of distal DoFs in a user’s prosthesis [59, 60]. Results from this study support that compensatory motions are not solely the result of a lack of DoFs in an upper-limb prosthesis or a lack of reliable control in the DoFs that are present. If so, we would expect the angular RoM between the CG_AR_ and EG to be the same during virtual prosthesis operation. Instead, we see that the EG consistently produces a larger angular RoM than the CG_AR_, even though both groups have the same distal DoFs and training errors (Fig. 9). An explanation for this kinematic difference is that compensatory movements are partially a consequence of the altered energetics of the limb-prosthesis system. Participants may support the external load of the prosthesis by altering their movement strategy to engage the more developed musculature of their trunk, chest, and upper arm. This explanation is further supported by the fact that the angular RoM for the CG_AR_ is greater in the pre- and post-test, when their limb is loaded, than during AR training, when their limb is unloaded, for each DoF. This is in contrast to the EG, whose members experienced limb loading during both the pre- and post-test and the AR training and did not present a difference in angular RoM between the physical and AR manipulation tasks. It is possible that a follow-up study with individuals using an osseointegrated prosthetic solution, in which the prosthesis load is supported by the user’s skeletal system, may provide more insight on the importance of external loads to compensatory movement strategies.

Secondary analysis also shows the effect limb loading has on a prosthesis user’s visuomotor behavior in virtual spaces. When comparing visuomotor behavior between the two participant groups, the EG produced a much higher mean fixation percentage on the hand during object transport than the CG_AR_: 90.37% vs 64.09%. Functionally, this shows that EG participants found themselves more often visually verifying that their virtual prosthesis continued stable grasping of the cylinder during object transport. One explanation for this difference in visual fixation between the two groups is that the load applied to a user’s limb draws more cognitive attention simply by being an external stimulus that the user is not habituated to. It is possible that for an experienced prosthesis user, the level of visual fixation during transport would reduce to that of the CG_AR_, even with limb loading.

Both the EG and the CG_AR_ visually fixated on their hand during object transport more frequently than prosthesis users performing object manipulations with a physical prosthesis (mean of 29.7% over two types of object manipulations [53]). One explanation for this difference between the virtual and the physical prosthesis fixation percentages could be the lack of haptic feedback in the grasping for the virtual case. Without haptic feedback, it is natural for a user to rely on visual information to assess the validity of an object grasp [61]. Although the physical prosthesis users did not use specific haptic interfaces in Hebert et al., the majority of the users were operating body-powered prostheses which provide grasp information through the tension of their Bowden cables. In fact, a further study showed that visual fixation can be reduced to normative levels when providing appropriate feedback using a physical myoelectric prosthesis with haptic interfaces [62].

Our study was not without limitations. Most apparently, the longitudinal study was not conducted with a population of LL participants. While previous literature has shown that IL participants construct similar motor strategies as individuals with LL when using a bypass prosthesis, the results of this study can be strengthened with an extension of this work that explicitly includes the target population [63]. Another limitation of this study is that general motor performance was characterized by only a single outcome measure: the PHAM. An extension of this work should include multiple functional outcome measures to better understand how limb loading enhances motor performance more generally [52, 64, 65].

Another limitation of this work was the location of the prosthesis relative to the limb. In the AR environment, the virtual prosthesis was mounted as an extension of the user’s limb, as if the user was wearing a traditional socket interface. In contrast, the physical prosthesis system mounted the prosthesis parallel to the operating arm, offset by 4 cm. This is due to a limitation of the AR system’s rendering pipeline. Because the AR system renders the real-world environment using stereoscopic camera pass-through, it is not possible to render a virtual object (like the prosthesis) behind a real-world object (like the intact limb). Doing so would produce significant visual artifacts at the boundaries of the overlapping real and virtual objects due to insufficient resolution of the depth data measured from the HMD.

It is unclear what effect this lack of offset may have had on a participant’s ability to embody the prosthesis. Page et al. claimed that prosthesis embodiment “depends on congruence (plausible anatomical orientation), temporal and spatial synchrony (between visual and proprioceptive or tactile feedback), and ‘bodily resemblance’” [66]. While not offsetting the virtual prosthesis increases congruence, it also decreases spatial synchrony with the socket’s proprioceptive feedback. However, literature does suggest that an offset, beside-the-hand configuration for a prosthesis simulator is biomechanically valid and does not have any significant effect on the joint kinematics of a user with an intact limb [67]. Nevertheless, an extension of this work should include subjective questionnaires and cortical imaging to understand the strength and progression of device embodiment in virtual training protocols.

A limitation of the training framework proposed in this study is that proprioceptive information was provided by physically loading the participant’s operating limb. As stated previously, the residual limb of a prosthesis user may be hypersensitive to physical loading in the time right after the surgery, when a prosthesis training intervention may be the most valuable [15, 16]. This hypersensitivity would make limb loading unfeasible. To overcome this limitation, proprioceptive information can be provided using less strenuous methods of joint-torque feedback [68, 69] or transcutaneous stimulation [70].

## Conclusion

This study investigated the effect that limb loading has on complex motor skill transfer from virtual to physical upper-limb prosthesis control. Participants who underwent an AR prosthesis training regimen with their operating limb loaded produced a significantly greater post-training increase in functionality when compared to participants who underwent the same training without the limb loading. Secondary analyses show that both compensatory motion and hand fixation percentage are increased when performing manipulations with the operating limb loaded, irrespective of prosthesis control capacity. Overall, the increased sensory fidelity afforded by limb loading helped to increase the level of skill transfer from an augmented reality prosthesis control task to an analog in physical reality.

## Data Availability

The data used in the current study are available from the corresponding author upon reasonable request.

## Abbreviations

AR: Augmented reality
LL: Limb loss
EMG: Electromyogram
DoF: Degree of freedom
PHAM: Prosthetic Hand Assessment Measure
HMD: Head-mounted display
MPL: Modular prosthetic limb
RE: Rest
HO: Hand open
HC: Hand close
WP: Wrist pronate
WS: Wrist supinate
LDA: Linear discriminant analysis
IL: Intact limb
CG_AR_: Augmented reality control group
CG_LD_: Load control group
EG: Experimental group
RoM: Range of motion
GaMA: Gaze and movement assessment
